# Development and regulation of pedicel abscission in tomato

**DOI:** 10.3389/fpls.2015.00442

**Published:** 2015-06-11

**Authors:** Yasuhiro Ito, Toshitsugu Nakano

**Affiliations:** Food Biotechnology Division, National Food Research Institute, National Agriculture and Food Research Organization, Tsukuba, Japan

**Keywords:** abscission, pedicel, MADS-box, ERF, tomato

## Abstract

To shed unfertilized flowers or ripe fruits, many plant species develop a pedicel abscission zone (AZ), a specialized tissue that develops between the organ and the main body of the plant. Regulation of pedicel abscission is an important agricultural concern because pre-harvest abscission can reduce yields of fruit or grain crops, such as apples, rice, wheat, etc. Tomato has been studied as a model system for abscission, as tomato plants develop a distinct AZ at the midpoint of the pedicel and several tomato mutants, such as *jointless*, have pedicels that lack an AZ. This mini-review focuses on recent advances in research on the mechanisms regulating tomato pedicel abscission. Molecular genetic studies revealed that three MADS-box transcription factors interactively play a central role in pedicel AZ development. Transcriptome analyses identified activities involved in abscission and also found novel transcription factors that may regulate AZ activities. Another study identified transcription factors mediating abscission pathways from induction signals to activation of cell wall hydrolysis. These recent findings in tomato will enable significant advances in understanding the regulation of abscission in other key agronomic species.

## Introduction

Similar to leaves, flowers and young fruits shed when the organs become unneeded or as a result of environmental stresses; for example, failure of pollination results in abscission of the unfertilized flowers. Also, in “June drop” in apple (*Malus* × *domestica*), some young fruitlets abscise at an early developmental stage (Bangerth, 2000). Just after flowering, apple trees often bear more fruits than they can support to maturity; thus the plants shed some fruits to limit fruit set. In addition, when fruits on a plant ripen, abscission of the fruits helps to disperse the seeds. The pedicel, a stem, or a stalk structure, connects at the base of the flower or fruit, attaching the organ to the plant body. In many species, an abscission zone (AZ) forms in the pedicel to enable regulated separation of the fruit or flower from the main plant body (Sexton and Roberts, 1982; Tabuchi et al., 2001; Roberts et al., 2002).

In agricultural applications, pedicel abscission is a critical trait directly affecting crop yields; thus the regulation of abscission has been important since ancient times. During the domestication of cereal crops such as rice (*Oryza sativa*), maize (*Zea mays*), or wheat (*Triticum aestivum*), early farmers selected for plants with reduced abscission (Doebley et al., 2006; Li et al., 2006; Lin et al., 2012). Cultivars carrying the trait conferring resistance to grain abscission retain the grain on the stalk, rather than dropping it on the ground.

Regulation of abscission also remains an important trait in modern breeding programs. Research in tomato has identified several mutations that block formation of the pedicel AZ, producing a “jointless” phenotype (Butler, 1936; Rick, 1967; Roberts et al., 2002), which has proven useful in tomato cultivars grown for industrial processing of tomato puree or juice. In these cultivars, fruits can be mechanically harvested without the pedicel and sepals because, in the absence of a breaking point in the pedicel AZ, the fruit detaches at the next breaking point, the calyx AZ at the proximal end of the fruit, and the green organs remains on the plant. This reduces the labor and time required to remove the pedicel and sepals during harvesting (Zahara and Scheuerman, 1988).

Fruit abscission is also an important trait for tree fruit production. In apple, abscission affects the fruit yield at several stages (Celton et al., 2014). The trees shed young fruitlets as “June drop,” as described above. Thinning of young fruits is an important practice to control fruit load and chemicals that induce partial abscission of fruit have been developed to reduce the labor required for thinning (Bangerth, 2000). After the early developmental stages, fruits at the expanding stage remain stably attached to the plant via the pedicels but the attachment gradually loosens during the initiation of ripening. However, severe weather can cause fruit to drop prematurely. For example, in Japan, the autumn fruit harvest coincides with the typhoon season and large numbers of fruits just before harvest time drop by the strong winds, which break a boundary between the plant body and pedicel, where the AZ is localized, resulting in severe damage to production (Yamamoto et al., 2012; Fujisawa et al., 2015).

## Pedicel AZ Structure and Development in Tomato

The AZ, a specialized tissue for organ abscission, forms at a predetermined site on the organ that will abscise. Anatomical studies revealed that an AZ includes several layers of small, densely cytoplasmic cells that forms at an early stage of pedicel development and proliferation of the cells is observed during fruit development (Addicott, 1982; Sexton and Roberts, 1982; Tabuchi and Arai, 2000; Patterson, 2001). These properties suggest that these cells may be arrested in an undifferentiated state (van Nocker, 2009). In tomato, initial differentiation of the pedicel AZ occurs when the flower sepal differentiates from the primordium. AZ cells first form in the inner region of the young pedicel and then the AZ structure gradually extends to the outer tissues (Tabuchi, 1999; Liu et al., 2014). The innermost cell layer has a critical role in AZ development, as examination of chimeric plants consisting of layers of jointless mutant cells and wild-type cells showed that the genotype of the inner layer (L3) determines cell fates of overlaying layers L1 (outer layer) and L2 (middle layer) and whether they differentiate into AZ tissue (Szymkowiak and Irish, 1999). At the flower anthesis stage, pedicel AZ tissues have developed into six to eight cell layers that extend across the pedicel. The AZ cells around the vascular tissue and cortex can still divide (Tabuchi and Arai, 2000), suggesting that the AZs in flower pedicels maintain meristem-like activity.

Normally, pedicel abscission is induced if flower fertilization fails or the fruit ripens fully. Pedicel abscission can also be induced artificially by flower removal (Roberts et al., 1984; Meir et al., 2010; Nakano et al., 2013) or ethylene treatment (Roberts et al., 1984; Wang et al., 2013); several studies have used these treatments to analyze abscission. Roberts et al. (1984) observed that cell separation for abscission first took place at the cortex within the distal side of the AZs if the pedicel was treated with ethylene. Also, Tabuchi et al. (2001) reported that pedicel abscission occurred first at the epidermis of the AZ if abscission was induced by emasculation. Dissolution of the middle lamella commonly occurred in response to either treatment, and cell wall hydrolysis enzymes and remodeling proteins, such as polygalacturonase (tomato abscission-related polygalacturonase; TAPG), endo-β-1,4-glucanase (also referred as cellulase; Cel), xyloglucan endotransglucosylase/hydrolase (XTH), and expansin, play a critical role in abscission (Roberts et al., 2002; Tucker et al., 2007; Cai and Lashbrook, 2008). The abscission-inducing treatment also caused enlargement of the epidermal cells in tomato (Tabuchi et al., 2001). Cell enlargement during abscission also occurs in other plant systems such as bean leaves (McManus et al., 1998) and *Arabidopsis* flower organs (Shi et al., 2011), and this enlargement may confer mechanical force to facilitate abscission (Shi et al., 2011). The abscised surface of the proximal side formed thickened and lignified cell walls, implying that a protective layer forms to prevent pathogen invasion (Tabuchi et al., 2001).

## MADS-Box Family Transcription Factors Regulate Pedicel AZ Development in Tomato

The most important breakthrough in abscission research was the identification of the *jointless* (*j*) mutant locus (Mao et al., 2000), which causes the plant to fail to develop pedicel AZs. The *j* locus was isolated by map-based cloning and the wild-type gene encodes a MADS-box transcription factor. In the same year, independent work on an early-flowering mutant identified *Arabidopsis SHORT VEGETATIVE PHASE* (*SVP*), which encodes a MADS-box protein with high similarity to J (Hartmann et al., 2000). Although J and SVP have high amino acid sequence similarity, they have distinct functions, with SVP acting as a repressor of the floral transition. Moreover, *Arabidopsis* plants do not shed fruits from the pedicels. Also, in several tree fruit species, *SVP* homologs may play roles in bud dormancy (Li et al., 2009; Yamane et al., 2011; Wu et al., 2012).

Further studies identified two additional tomato MADS-box genes regulating pedicel AZ development, *Macrocalyx* (*MC*) and *SlMBP21*. *MC* was originally identified in a study of *rin* (*ripening inhibitor*), which regulates fruit ripening. The *rin* mutation produces non-ripening fruits with large sepals (Vrebalov et al., 2002). The cloning study identified two nearby genes, *RIN* and *MC*, both of which encode MADS-box genes. *RIN* regulates ripening and *MC* regulates sepal size (Vrebalov et al., 2002). The *rin* mutation also shows a weak effect on pedicel AZ development and antisense-mediated knockdown revealed that *MC* also plays a role in pedicel AZ development (Nakano et al., 2012). A comprehensive interaction study of tomato MADS-box proteins using yeast two-hybrid system initially identified SlMBP21 as a MADS-box protein interacting with J (Leseberg et al., 2008). A gene knockdown study revealed that *SlMBP21* also participates in pedicel AZ development (Liu et al., 2014). These studies showed physical interactions among J, MC, and SlMBP21, suggesting that these three MADS-box proteins form a complex. At an early stage of AZ initiation, these MADS-box genes are co-expressed in vascular tissue derived from the L3 layer required for AZ development (Szymkowiak and Irish, 1999; Liu et al., 2014). In *Arabidopsis*, the J homolog SVP and the MC homolog AP1 likely form a dimer as an active form to regulate floral identity (Gregis et al., 2009). SEP family proteins, including SlMBP21, play an important role in forming multimers of MADS-box proteins by acting as a glue (Immink et al., 2009). Thus, multimer formation of J, MC, and SlMBP21 may be a conserved activity among plant species, although the targets of biological regulation by homologous MADS-box proteins may differ in each plant.

Is the regulation of pedicel AZ development by the MADS-box transcription factors conserved in other plant species, or is it specific to tomato? Ectopic expression of the apple SVP family MADS-box gene *MdJb* in a tomato *j* mutant restored the formation of pedicel AZ structure in the *j* mutant (Nakano et al., 2015). The restored AZs showed abscission-associated expression of cell wall hydrolysis enzyme genes and complete pedicel abscission, as in wild-type tomato plants. The results suggest that the regulation of pedicel AZ development in plants by the MADS-box transcription factors may be conserved, but other plant systems remain to be examined. Further investigation will be required to understand the mechanism of AZ development in other plant species.

## Genes Expressed in Tomato Pedicel AZs

Before abscission, pedicel AZs attach the flowers firmly to the plant body, but when the AZ cells perceive an abscission-stimulating signal, the adhesion immediately starts to loosen. During abscission, the gene expression pattern in the AZ changes drastically; genes for cell wall hydrolysis enzymes, such TAPG and Cel, and for factors regulating programmed cell-death increase intensely and specifically at the AZ (Roberts et al., 2002; Cai and Lashbrook, 2008; Meir et al., 2010; Bar-Dror et al., 2011). In addition to these genes, a transcriptome study during initiation of abscission found many genes possibly responsible for regulatory roles in abscission, such as genes for transcription factor families of ARF, Aux/IAA, KNOX, HAT, bHLH, AP2, NAC, AGL, and WRKY, genes for components of signal transduction pathways such as a LRR-RLK and a Ser/Thr protein kinase, and a gene for a component of a RNA-induced silencing complex, AGO1 (Meir et al., 2010). The analyses also provided specific expression patterns of phytohormone-related genes, which confirmed and improved a conventional abscission-inducing model with the substantial evidence (Patterson, 2001; Roberts et al., 2002; Meir et al., 2010); a decrease in auxin provides the first signal for abscission, and reactions to the decrease in auxin, including down-regulation of genes induced by auxin (such as *Aux/IAA* genes and other transcription factor genes) and up-regulation of genes repressed by auxin, confers ethylene-sensitivity and abscission competence to the AZ. Then increased ethylene production, due to the up-regulation of genes for ethylene biosynthesis (such as *ACS*, encoding 1-aminocyclopropane-1-carboxylate (ACC) synthase), leads to AZ-specific up-regulation of the genes for abscission, such as genes encoding cell wall-modifying proteins and pathogenesis-related proteins, development of a protective layer on the surface of the abscised tissue, and so on.

Before the onset of pedicel abscission, the plant maintains firm cell-to-cell adhesion at the AZs to allow continuous growth from the flower to the mature fruit. To maintain the adhesion and the competence to react to an abscission-inducing signal, the AZ cells might undergo specific regulation. A transcriptome analysis comparing gene expression between pedicel AZs and the flanking pedicel regions at anthesis (Nakano et al., 2013) identified about 90 genes specifically expressed in AZ cells, including genes for transcription factors, phytohormone-related proteins, cell wall modification enzymes, lipid metabolism, and others. Most interestingly, the AZ-specific gene set included transcription factor genes that encode key regulators of meristem-associated functions, including a tomato homolog of *WUSCHEL* (*LeWUS*), *GOBLET* (*GOB*), *LATERAL SUPPRESSOR* (*Ls*), and *Blind* (*Bl*). *WUS* expressed in *Arabidopsis* shoot apex is required for maintenance of stem cells in an undifferentiated state (Mayer et al., 1998). *GOB* is a member of the NAC family transcription factor genes and its *Arabidopsis* homolog genes, *CUP-SHAPED COTYLEDONs* (*CUCs*), are involved in shoot meristem formation and specification of organ boundaries (Aida et al., 1997; Blein et al., 2008; Berger et al., 2009). *Ls* and its *Arabidopsis* homolog are known to regulate axillary meristem initiation (Schumacher et al., 1999; Greb et al., 2003). *Bl* and its *Arabidopsis* homolog of *REGULATOR OF AXILLARY MERISTEM* (*RAX*) also involved in axillary meristem formation (Schmitz et al., 2002; Keller et al., 2006). These transcription factors were suppressed in the pedicels of AZ-deficient plants, the *j* mutant, and *MC-* and *SlMBP21-*suppressed plants (Nakano et al., 2012; Liu et al., 2014). Also, *LeWUS*, *GOB*, and *Ls* were down-regulated immediately after an abscission-inducing treatment while *Bl* was up-regulated (Nakano et al., 2013). These characteristic expression patterns suggest that these transcription factor genes play important roles in the AZs. Similar to meristems, AZs include small cells that likely exist in an undifferentiated state (van Nocker, 2009); thus, these transcription factors may regulate the maintenance of these undifferentiated cells in both tissues. In rice flower pedicels, homologs of *Bl*, *GOB*, and *Ls* are expressed specifically in the AZs, indicating that the mechanism of regulation by these transcription factors may be conserved in monocots and dicots (Nakano and Ito, 2013).

A recent study showed an intriguing result on the undifferentiated properties of the AZ cells. Constitutive expression of a miRNA-resistant form of a tomato homolog of the *REVOLUTA* gene, encoding a Class III homeodomain-leucine zipper (HD-ZIP III) transcription factor, caused the transgenic plants to produce ectopic flowers from the pedicel AZs (Hu et al., 2014). In the AZs at anthesis, the transgenic plants expressed *Bl* and *GOB* at significantly higher levels than the wild-type plants. The results imply that pedicel AZs include undifferentiated cells that have the potential to develop into flower primordia, and the transcription factors expressed in the AZs may coordinately regulate the maintenance and proliferation of the undifferentiated AZ cells.

These transcriptome analyses identified genes specifically expressed in tomato pedicel AZs, and of them, two transcription factor genes, *SlERF52* and *KD1*, were further analyzed for their effect on AZ functions, as described in the next section.

## Transcription Factors Connecting Abscission-Inducing Signals and Abscission Processes

Of the transcription factor genes expressed in tomato pedicel AZs, the ERF family transcription factor gene *SlERF52* was further investigated by RNAi-mediated knockdown assays (Nakano et al., 2014). The *SlERF52*-knockdown plants developed pedicel AZ structures similarly to wild-type plants; however, the responses to an abscission-inducing treatment differed in the knockdown and wild-type plants. In wild-type plants, removing the anthesis-stage flower from the pedicel usually induces pedicel abscission within 2 days; in the *SlERF52*-knockdown plants, pedicel abscission took significantly longer. The knockdown disturbed the abscission-specific up-regulation of the genes for hydrolytic enzymes, such as TAPG and Cel, indicating that the suppression of the hydrolytic enzymes caused the delay in abscission. The result suggests that the SlERF52 ERF transcription factor functions as a component of a signaling pathway for pedicel abscission and plays a key role in the induction of expression of genes involved in cell wall hydrolysis. The induction of the hydrolytic enzyme genes during abscission, however, may require an additional factor to activate SlERF52. The expression levels of *SlERF52* did not differ before and after the abscission-inducing treatment; thus, the expression level of *SlERF52* cannot explain the activation of abscission. On the other hand, before the onset of abscission, the AZ-specific expression of *LeWUS*, *Ls*, and *GOB* requires *SlERF52*, implying that SlERF52 acts before and during abscission, but the transcriptional targets of SlERF52 apparently differ in the two stages. Explaining the functional switching of *SlERF52* may require additional factors, such as stage-specific co-factors of SlERF52 or repressor proteins at SlERF52-binding sites. The identification of the switching mechanism will provide further insights into the regulation of pedicel abscission.

Another transcription factor gene expressed specifically in the tomato pedicel AZ, *KD1*, a *KNOTTED1-LIKE HOMEOBOX* (*KNOX*) family gene, was investigated for function in pedicel and petiole AZs (Ma et al., 2015). Down-regulation of *KD1* significantly delayed abscission of the pedicel and even petiole and up-regulation of *KD1* promoted abscission. The investigation suggested that *KD1* controls abscission by regulating genes that modulate auxin levels (Ma et al., 2015). Identification of the regulator of abscission in both pedicels and petioles provides substantial evidence that abscission in these tissues involves identical regulatory mechanisms, in contrast to their distinct mechanisms regulating AZ development (Szymkowiak and Irish, 1999). Further investigation of the relationship between SlERF52 and KD1 may reveal their activities in abscission processes more clearly.

## Conclusion

**FIGURE 1:**
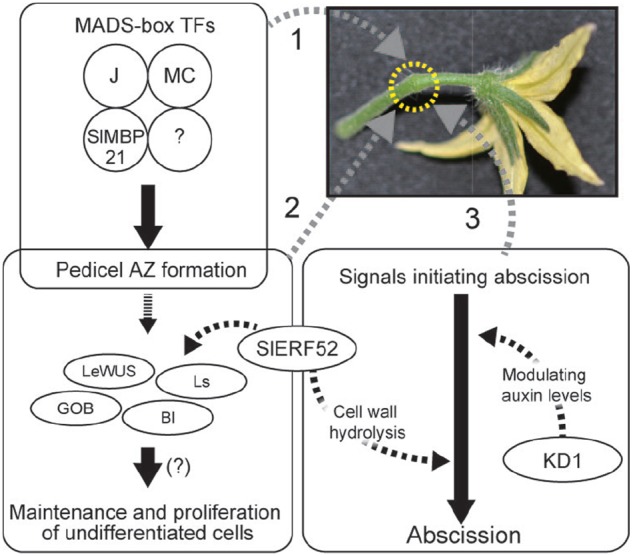
**Regulation of pedicel AZ functions.** 1. MADS-box proteins form tetramers and regulate pedicel AZ formation. 2. Undifferentiated cells are maintained in the pedicel AZ. AZ-specific transcription factors may be involved in the maintenance and proliferation of the undifferentiated cells. Expression of these transcription factor genes requires the activity of SlERF52. 3. In response to abscission-initiating signals, KD1 and SlERF52 activate abscission by modulating auxin levels and up-regulating genes encoding cell wall hydrolysis enzymes, respectively.

These recent advances in our understanding of the regulation of pedicel abscission revealed key factors involved in AZ development and signal transduction in the initiation of abscission. Figure 1 shows a current model of development of AZs and induction of abscission in tomato pedicels. The MADS-box transcription factor complex regulates pedicel AZ development. The developed AZ contains undifferentiated cells, probably maintained by a mechanism similar to that found in meristems. The signals of decreased auxin and increased ethylene induce abscission and SlERF52 and KD1 possibly connect the phytohormone signaling pathway and abscission processes. A remaining mystery is another tomato jointless mutation, *jointless-2* (*j-2*), which is the best used mutation in practical breeding programs of processing tomatoes. A candidate gene for the mutation was reported but it has not been fully identified yet (Yang et al., 2005).

The outline of the current model constructed in tomato will facilitate further detailed studies on pedicel functions in tomato and other plants and these studies will provide new applications for fruit crops to improve their productivities.

### Conflict of Interest Statement

The authors declare that the research was conducted in the absence of any commercial or financial relationships that could be construed as a potential conflict of interest.
